# Prevalence of sickness presenteeism and associated factors among primary school teachers in Gondar city, northwest Ethiopia

**DOI:** 10.3389/fpubh.2024.1384325

**Published:** 2024-08-09

**Authors:** Yimer Mamaye, Dawit Getachew Yenealem, Molla Fentanew, Tadiwos Abebaw, Christian Melaku, Anmut Endalkachew Bezie, Alebachew Bitew Abie, Amensisa Hailu Tesfaye

**Affiliations:** ^1^Department of Occupational Health and Safety, College of Medicine and Health Sciences, Wollo University, Dessie, Ethiopia; ^2^Department of Environmental and Occupational Health and Safety, Institute of Public Health, College of Medicine and Health Sciences, University of Gondar, Gondar, Ethiopia; ^3^Department of Physiotherapy, School of Medicine, College of Medicine and Health Sciences, University of Gondar, Gondar, Ethiopia

**Keywords:** sickness presenteeism, health at work, primary school, teachers, Ethiopia

## Abstract

**Background:**

Sickness presenteeism, the phenomenon of people going to work despite being ill, is an occupational and psychosocial condition that hurts both the health of workers and organizational productivity. It negatively affects health, increases health-related costs, and the risk of contagious diseases. Primary school teachers are particularly vulnerable to this problem, although little is known about its scope and associated factors. This study aimed to determine the prevalence and factors associated with sickness presenteeism among school teachers in Northwest Ethiopia.

**Methods:**

An institution-based cross-sectional study was employed and the study period was from April 18 to May 18, 2023. A sample of 633 primary school teachers was recruited using two-stage stratified random sampling. Data were collected using structured self-administered questionnaires. Epi-data version 4.6 and STATA version 14 were used for data entry and analysis, respectively. Binary logistic regression analysis was used. A multivariable logistic regression model with an adjusted odds ratio was fitted for statistical significance.

**Results:**

A total of 603 school teachers participated in this study, with a response rate of 95.26%. The overall prevalence of sickness presenteeism in the last 12 months was 54.7% (*N* = 330) [95% CI (50.9, 58.7)]. Private school teachers [AOR: 2.21, 95% CI (1.14, 4.28)], low supervisor support [AOR: 1.53, 95% CI (1.06, 2.20)], lack of staff replacement availability [AOR: 2.74, 95% CI (1.85, 4.06)], low colleague support [AOR: 2.17, 95% CI (1.40, 3.37)], unsuitable household conditions [AOR: 1.49, 95% CI (1.08, 2.34)], and strict attendance control [AOR: 2.54, 95% CI (1.67, 3.85)] were factors significantly associated with sickness presenteeism.

**Conclusion:**

The prevalence of sickness presenteeism was relatively high among primary school teachers because of factors such as low support from supervisors and colleagues, strict attendance control, lack of staff replacement, unsuitable household conditions, and private school type. Strategies to promote teachers’ health include fostering a culture of support and collaboration among colleagues, recruiting adequate staff, and implementing liberal attendance policies.

## Introduction

Sickness presenteeism (SP), the phenomenon of people going to work despite being ill, is an occupational and psychosocial condition that harms both the health of workers and organizational productivity (1, 2). It is often seen as an act of organizational citizenship and a sign of commitment and loyalty to employers and colleagues (3, 4). However, working while sick can delay recovery, increase the risk of future health problems and absenteeism, hamper productivity, and lead to errors, accidents, and injuries (5).

Sickness presenteeism (SP) is a global phenomenon affecting employees, organizations, and society (6). Sickness Presenteeism among school teachers can lead to work-related stress, depression, fatigue, exhaustion, burnout, and serious physical health conditions like coronary artery disease (7–12). Frequent practice of sickness presenteeism has been linked to the spread of communicable diseases, which can circulate in workplaces and contribute to pandemics (13–15). Working while ill can also negatively impact team dynamics and morale, as employees may perceive underperformance as a lack of competence and motivation, leading to negative effects on team performance. Additionally, sickness presenteeism among school teachers can indeed harm student’s developmental and learning outcomes. When teachers come to work while they are ill, it can affect their ability to provide quality instruction and individualized attention to students (5, 16, 17).

Evidence in the literature analyzing the effects of presenteeism observed in cost estimation studies and economic evaluations showed that presenteeism costs exceed absenteeism costs (18). According to the Harvard Business Review, the total estimated cost of presenteeism in the United States was more than $150 billion per year, and its economic cost was far greater than that of sickness absenteeism or disability (16). In Australia, the cost of presenteeism incurred by the Australian economy due to teachers attending work while experiencing health issues is estimated to be AUD 8092 per teacher, indicating the economic burden associated with presenteeism and its effects on workforce efficiency and productivity within the educational sector (19). Apart from the financial implications for the company, employee presenteeism poses a danger to the physical and emotional well-being of teachers and students (20). Regarding this adverse attribute, presenteeism may result in learning difficulties, mental health problems in students, an unhealthy relationship with students, and the loss of the ability to be a role model for students (21, 22).

Sickness Presenteeism (SP) is becoming a growing health problem in the workplace, and education is one of the most negatively affected sectors (23). In a recent study, 46% of education workers reported working while sick whereas 44% of those in the care and welfare sector reported doing so (24). Preschool teachers were the most likely to report sickness presenteeism, with 90% of them saying they had come to work while sick in the past year. Primary school teachers were next, with 73% reporting SP (25). The prevalence of SP among school teachers varies greatly between nations. A study conducted on German teachers revealed sickness presenteeism incidence of 57.1% (26); Sweden, 55% (27), Leon, Nicaragua, 65.2% (25); and Brazilian, 43% (28).

Literature explores various theoretical framework models to explain the variation in presenteeism prevalence across nations. The variation in SP prevalence across nations may be attributed to several factors, such as sociodemographic factors including age, sex, marital status, income, and education (20, 29, 30), and behavioral factors such as treating work as home, being over-committed to work, conservative attitude towards absence, alcohol consumption, and physical exercise lead to presenteeism (31, 32). Furthermore, evidence in the literature used the job-demand-resource (JD-R) theory and effort-reward imbalance model to associate sickness presenteeism and various work-related factors such as job demand, workload, time pressure, job stress, job insecurity, job control, lack of staff availability, attendance pressure, and job satisfaction influence the decision to experience sickness presenteeism (31, 33, 34).

Although most organizations are taking steps to measure and manage sick leave, the prevalence and risk factors for SP are largely overlooked (35). Organizations can encourage a culture of SP, as someone who works sick is often viewed as an ideal employee who dedicates and commits to his or her employer (36). Such behavior may also be reinforced in organizations that reward employees with exemplary attendance, use medical records as a criterion for promotion, or stigmatize or penalize employees whose attendance is deemed unsatisfactory (37). Intense and widespread experiences of sickness presenteeism among primary school teachers lead to failure in classroom management, less time spent on tasks during the days when the teacher is present, a loss of moral support, a reduction in performance, and decrease in the quality of education (33).

The concept of occupational health in developing countries, including Ethiopia, is still young. Employees have the right to be healthy in the workplace. The issue of sickness presenteeism among primary school teachers is a pressing concern, but its scope is unclear due to limited information on health problems and their associated factors in the country. Measuring sickness presenteeism would provide a clear picture of teachers’ health. In addition, it is important to identify and address the factors that contribute to sickness presenteeism to improve the health and well-being of teachers and to ensure that they can provide their students with the best possible education. Therefore, to fill this gap, the present study aimed to determine the prevalence of sickness presenteeism and associated factors among primary school teachers in Gondar city, Northwest Ethiopia. The findings of this study provide evidence for policymakers, educational leaders, and researchers to help them identify school interventions that improve the health of teachers and learning outcomes and promote the best teaching-learning environment possible.

## Materials and methods

### Study design, period, and setting

An institution-based cross-sectional study was conducted among primary school teachers in Gondar city and the study period was from April 18 to May 18, 2023. In 2019, the city’s total population was estimated to be 500,788, of whom 300,000 were males. In 2023, based on the information obtained from the Gondar city administration education bureau, in the city, there are 65 primary schools (44 public primary schools and 21 private primary schools). Among these schools, there were 1973 primary school teachers (1,470 public primary school teachers and 503 private primary school teachers).

### Source and study populations

The source populations were all public and private primary school teachers in Gondar city. On the other hand, all public and private primary school teachers working in the selected schools were considered to constitute the study population.

### Inclusion and exclusion criteria

All primary school teachers who were working in public and private schools in Gondar city for at least 1 year prior to the study were included. However, teachers who were on annual, maternity, and sick leave during the data collection period were excluded.

### Sample size determination and sampling technique

The sample size was calculated using a single population proportion formula (38) by considering the following statistical assumptions: *Z*α/2 = the value of the standard normal curve score corresponding to the given confidence interval (CI); proportion (*p*) of sickness presenteeism among school teachers = 50% since there has been no previous study in the country in a similar context; and margin of error (*d*) = 5%. The single proportion formula was used: $n={\left(Z\alpha {/}_{2}\right)}^{2}\;\frac{\left[p\;\left(1-p\right)\right]}{d^{2}}$ , where n is the initial sample size, *Z* is 1.96, the corresponding *Z* score is the 95% CI, p is the proportion of sickness presenteeism, which is 0.5, and d is the margin of error, which is 0.05. Then, $n={\left(1.96\right)}^{2}\;\frac{\left[0.5\;\left(1-0.5\right)\right]}{0.05^{2}}$ = 384. Assuming a 10% response rate, 384 + 38 = 422. When considering the design effect, the calculated sample size was multiplied by the design effect to correct for the estimated sampling variance. Therefore, the overall sample size for this study was (384 + 38) *1.5 = 633 primary school teachers.

**Figure 1:**
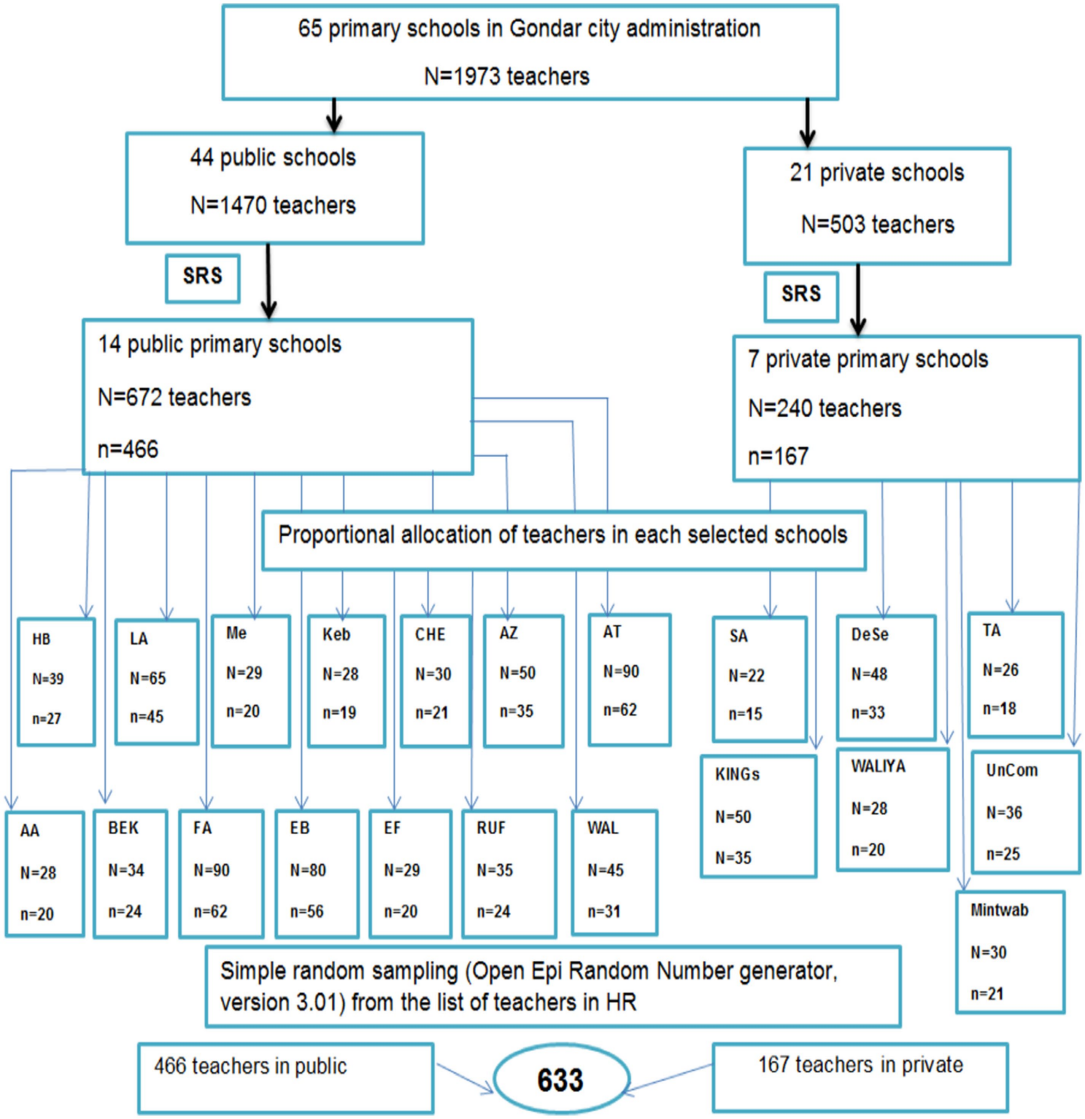
Schematic presentation of the sampling procedure for the prevalence of sickness presenteeism and associated factors among primary school teachers in Gondar city, Northwest, Ethiopia, 2023. Keys: HB = Hibret, LA = Leul-alemayehu, me = Meseret, Kebele10, CHE = chechela, AZ = Azezo, AT = Ayer-tena, AA = Addis-alem, BEK = Atse-Bekafa, FA = Atse-Fasil, EB = Ewuket-Ber, EF = Ediget-Feleg, RUF=Kilil-rufael, WAL = Walaj, AS = abbune-Samuel, DeSe = Debre Selam, TA = Tiwuld-Amare, UnCom = University community.

A two-stage stratified random sampling technique was used to recruit study participants. First, stratification was performed based on the ownership of the schools as public (44 schools) and private (21 schools). To ensure representativeness, 30% of each primary school was considered, e.g., 30%* 44 public ≈14 and 30%*21 private≈7. The required schools were selected by the lottery method. The calculated sample was proportionally allocated to all the selected schools. Finally, study participants were selected randomly using the Open Epi Random Program Version 3.01 (39) from the designated schools using the list of teachers obtained from the human resource department as a sampling frame (Figure 1).

### Operational definitions

#### Sickness presenteeism

Going to work despite feeling sick or sick at work was measured by using an item: In the previous 12 months, have you gone to work while feeling sick, even though it would have been truly reasonable to take sick leave? Yes/no. Teachers who indicated that they went to work despite their sickness in the past 12 months two or more times were considered to be experiencing sickness presenteeism (20, 26, 27, 37, 40–45).

#### Primary school teachers

Teachers who are assigned a grade ranging from 1 to 8.

#### Job stress

Measured by 8 items with responses on a five-point Likert scale ranging from 1 = never to 5 = always. The total score for each teacher was calculated by summing the scores for all the responses. The sum was dichotomized through a stress scale of 16 and above and was categorized as stressed (46, 47).

#### Over-commitment

Personality characteristic that makes it difficult for people to say no to other people’s wishes and demands and is measured by 6 items with responses on a five-point Likert scale ranging from 1 = strongly disagree to 5 = strongly agree (Chronbach’s alpha, α = 0.76). The total score for each teacher was calculated by summing the scores for all the responses. The sum was categorized with an average score of 22 and above and was considered to indicate that the patient was over-committed to his/her job (31, 48, 49).

#### Financial problems

Financial difficulties were measured by 4 items with responses on a five-point Likert scale ranging from 1 = strongly disagree to 5 = strongly agree (Chronbach’s alpha, α = 0.78). The total score for each teacher was calculated by summing the scores for all the responses. The sum of the scores was categorized at a median of 14 and above and was considered to indicate financial struggle (25).

#### Colleague support

This variable was measured by 4 items with a seven-point Likert scale ranging from 1 = strongly disagree to 7 = strongly agree (Chronbach’s alpha, α = 0.83). The sum was dichotomized into low/high by an average score of 18, and above was considered high colleague support (50).

#### Supervisor support

This variable was measured by 3 items on a five-point Likert scale ranging from 1 = very low to 5 = very high. The total score for each teacher was calculated by summing the scores for all the responses. The sum was dichotomized into low/high by a median score of 11, and above was categorized as high supervisor support (50).

### Data collection tools

The data were collected using a self-administered technique, and we applied a structured questionnaire, which was adapted from several studies regarding sickness presenteeism (25, 26, 42, 51, 52). The questionnaire is divided into five sections. The first part included socio-demographic characteristics such as sex, age, marital status, educational status, monthly salary, and work experience. The second section encompasses the prevalence of sickness presenteeism (yes/no) (20, 25–27). The third section contains work-related factors measured by the Copenhagen Psychosocial Questionnaire (COPSOQ), such as job satisfaction, job stress, staff replacement availability, daily teaching hours, job control, job demand, colleague support, supervisor support, job insecurity, social support, recognition, attendance pressure, school type, over-commitment, and teacher-student relationships (53). The fourth section focuses on socioeconomic factors such as household conditions, school infrastructure, and financial problems (50), and the last section encompasses behavioral factors such as physical exercise, khat chewing, alcohol consumption, cigarette smoking, and body mass index (BMI) (28). Overall, during the data collection, the study participants were recruited from the selected schools during their working hours, especially during break times and free periods.

### Data collection procedure

The data were gathered using a pretested and structured Amharic version of a self-administered questionnaire after ethical clearance was obtained from the Institutional Ethical Review Board (IERB) of the University of Gondar and informed written consent was obtained from the study subjects. Based on the pretest, necessary modifications were made to the questions, and participants involved in the pretest were excluded from the actual data analysis. Two days of training were given to the data collectors. The data were collected by trained data collectors.

### Data quality control

To ensure consistency, the questionnaire was developed in English first and then translated into Amharic and back into English by the authors with the assistance of language experts. Second, we employed two data collectors and one supervisor with prior experience and knowledge of the data collection process. The data collectors and supervisor had 2 days of training and orientation before the actual data collection on issues relating to the clarity of the questions, the objectives of the study, the confidentiality of the information, and the informed consent, as well as the roles and responsibilities maintained by both the data collectors and the supervisor during the data collection process. The principal investigator supervised both the data collectors and the supervisor. Third, we conducted a pretest 1 week before the actual data collection period on 5% (32) of the sample of Tedda primary school teachers, near Gondar city, to check the response, language clarity, appropriateness, and consistency of the instrument. Based on the findings from the pretest analysis, we modified some words and misinterpretations, minimized the number of questions, and corrected the ambiguous questions. The pretested data were not included in the actual data analysis. Problems faced during the data collection process were resolved by discussion on the spot with the principal investigator, supervisor, and data collectors.

### Data management and statistical analysis

The collected data were entered in EPI Data version 4.6, cleaned, and coded for analysis using STATA version 14. Descriptive statistics were carried out and presented with narration, tabulation, and graphical presentation. The normality, outliers, and multicollinearity of the variables were checked before running the bivariable and multivariable binary logistic regression analyses. The multicollinearity assumption was checked through the variance inflation factor (VIF), and all variables had a VIF < 5. A binary logistic regression (bivariable and multivariable binary logistic regression) analysis was performed to identify statistically significant variables.

The variables that had statistically significant associations (*p*-value <0.2) with the dependent variable in the bivariable logistic regression analysis were further considered candidates for multivariable logistic regression to control for the possible effect of confounding variables. Finally, statistically significant variables were established at *p*-values <0.05 in a multivariable binary logistic regression model, and an adjusted odds ratio (AOR) with a 95% confidence interval (CI) was used to measure the strength of the association. The Hosmer–Lemeshow test (at *p*-value>0.05) was used to determine the goodness-of-fit of the final models, and the results revealed that the model was well-fitted (*p* = 0.283).

## Results

### Socio-demographic characteristics of the study participants

A total of 603 out of 633 teachers participated in the study, with a response rate of 95.26%. Among the total participants, 344 (57%) of the teachers were female. The mean (±SD) age of the teachers was 34.94 (± 9.22) years. More than half of the teachers, 325 (53.9%) were married. Regarding educational level, 363 (60.2%) of the teachers had a bachelor’s degree. Nearly half (298, 49.1%) of the teachers had a monthly salary of less than 8,000 ETB (Table 1).

**Table 1:** Sociodemographic characteristics of primary school teachers, Northwest Ethiopia, 2023 (*n* = 603).

Characteristics	Categories	(*n*)	(%)
Sex	Male	259	43.0
	Female	344	57.0
Age	18–30	246	40.8
	31–45	273	45.3
	46–60	84	13.9
Marital status	Single	222	36.8
	Married	341	56.6
	Other^**#**^	40	6.6
Educational level	Diploma	226	37.5
	Bachelor’s degree and above	377	62.5
Monthly salary (ETB)	<8,000	296	49.1
	≥8,000	307	50.9
Teaching experience in years	1–10	308	51.1
	11–20	170	28.2
	21 and above	125	20.7

Keys: ^#^Divorced/Widowed; ETB, Ethiopian birr.

### Behavioral characteristics of the participants

Of all the participants, only 40 (6.6%) drank alcohol, and 13 (2.15%) drank alcohol three or more times a week. In addition, approximately 10 (1.7%) of the teachers were cigarette smokers. Of those who chewed khat, 6 (1.02%) were considered khat chewers. Regarding physical activity, 133 (22.1%) of the participants performed physical exercise at least two times a week (Table 2).

**Table 2:** Behavioral characteristics of primary school teachers, Northwest Ethiopia, 2023 (*n* = 603).

Characteristics	Categories	(*n*)	(%)
Alcohol drinking	Yes	40	6.6
	No	563	93.4
Drinking frequency	<3 days/week	27	4.45
	≥3 days/week	13	2.15
Cigarette smoking	Yes	10	1.7
	No	593	98.3
Khat chewing	Yes	13	2.2
	No	590	97.8
Chewing frequency/week	<3 days/week	7	1.18
	≥3 days/week	6	1.02
Physical exercise	Yes	133	22.1
	No	470	77.9
Body mass index (BMI)	Underweight	42	7.0
	Normal	478	79.3
	Overweight	83	13.7

### Work-related characteristics of participants

Out of the total participants, 436 (72.3%) were public school teachers. More than half of the teachers, 365 (60.5%) had low job demand, and 292 (48.4%) had low supervisor support. Of the total participants, 225 (37.3%) experienced job stress; almost half, 293 (48.6%) were satisfied with their jobs; and half of the teachers, 302 (50.1%) had good relationships with their students. In terms of staff replacement availability, 285 (47.3%) of the teachers stated that staff should not be replaced during illness absence. Moreover, 424 (70.3%) had strict attendance control due to sickness absence from supervisors. Furthermore, among the total participants, nearly half; 294 (48.8%) had low colleague support (Table 3).

**Table 3:** Work-related characteristics of primary school teachers, Northwest Ethiopia, 2023 (n = 603).

Characteristics	Categories	(*n*)	(%)
School type	Private	167	27.7
	Public	436	72.3
Daily teaching hrs.	≤4 h.	417	69.2
	>4 h.	186	30.8
Job demand	Low	365	60.5
	High	238	39.5
Recognition	Yes	200	33.2
	No	403	66.8
Supervisor support	Low	292	48.4
	High	311	51.6
Social support	Low	218	36.2
	High	385	63.8
Job security	Yes	310	51.4
	No	293	48.6
Job stress	Stressed	225	37.3
	Not stressed	378	62.7
Job satisfaction	Satisfied	293	48.6
	Not satisfied	310	51.4
Over-commitment	Overcommitted	210	34.8
	Not overcommitted	393	65.2
Strict control of attendance	Yes	424	70.3
	No	179	29.7
Job control	Low	263	43.6
	High	340	56.4
Colleague support	Low	294	48.8
	High	309	51.2
Teacher student relationship	Good	302	50.1
	Poor	301	49.9
Lack of staff replacement availability	Yes	285	47.3
	No	318	52.7

### Socioeconomic characteristics of the participants

Among the total participants, 247 (41%) had financial problems, and more than half, 330 (54.7%) of the teachers stated that their school infrastructure was poor. Moreover, 362 (60%) of the teachers’ households were unsuitable for resting during illness (Table 4).

**Table 4:** Socioeconomic characteristics of primary school teachers, Northwest Ethiopia, 2023 (*n* = 603).

Characteristics	Categories	(*n*)	(%)
Financial problem	Yes	247	41.0
	No	356	59.0
School infrastructure	Poor	330	54.7
	Good	273	45.3
Household for resting	Not suitable	362	60.0
	Suitable	241	40.0

### Prevalence of sickness presenteeism

**Figure 2:**
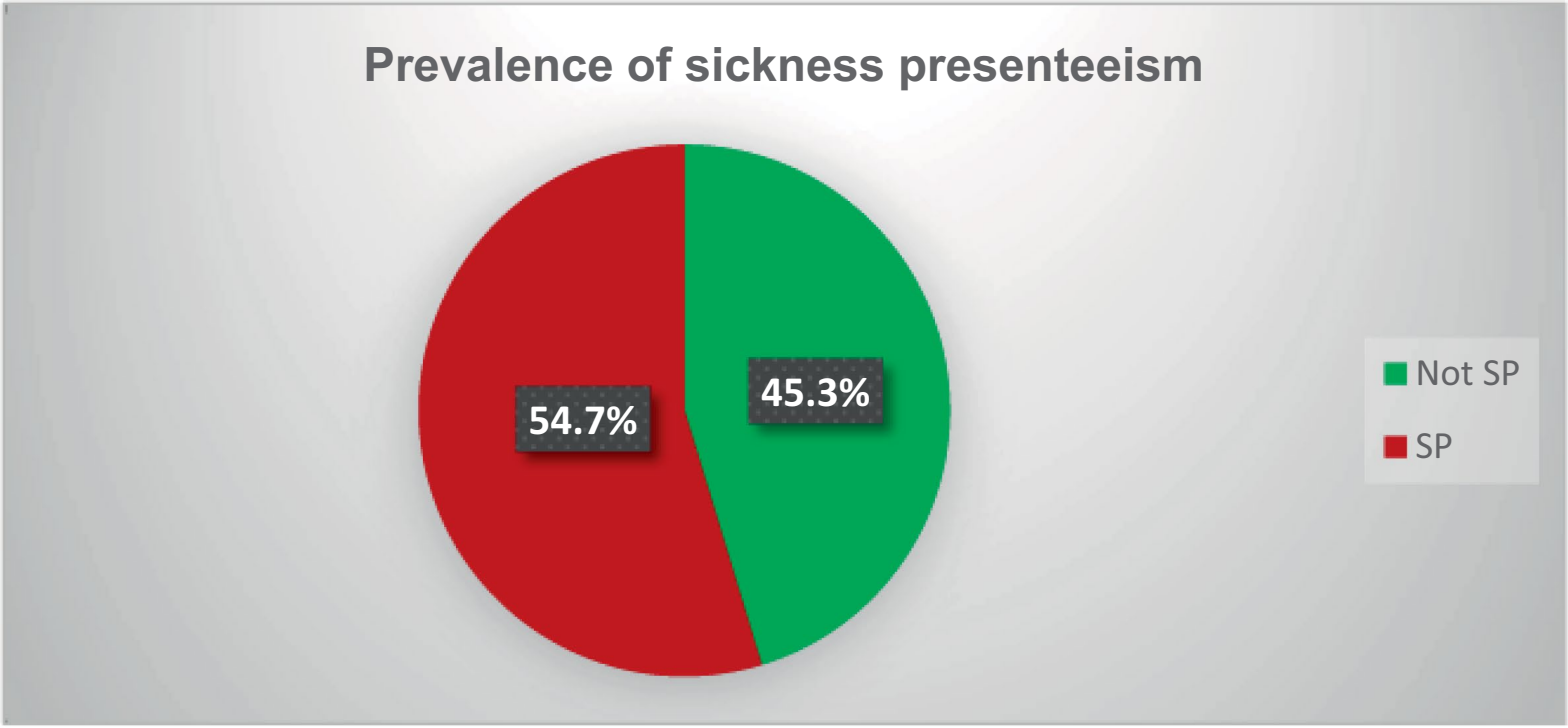
Pie chart showing prevalence of sickness presenteeism among primary school teachers in Gondar city, Northwest Ethiopia, 2023 (*n* = 603). Keys: SP = sickness presenteeism.

This study revealed that the overall prevalence of sickness presenteeism experienced over the past 12 months among primary school teachers in the study area was 54.7% (*n* = 330) [95% CI (50.9, 58.7)]. Of these, 255 (42.3%) had experienced SP between two and five sick days, and the remaining 75 (12.4%) had more than five sick days (Figure 2).

### Reported health conditions in relation to sickness presenteeism

**Figure 3:**
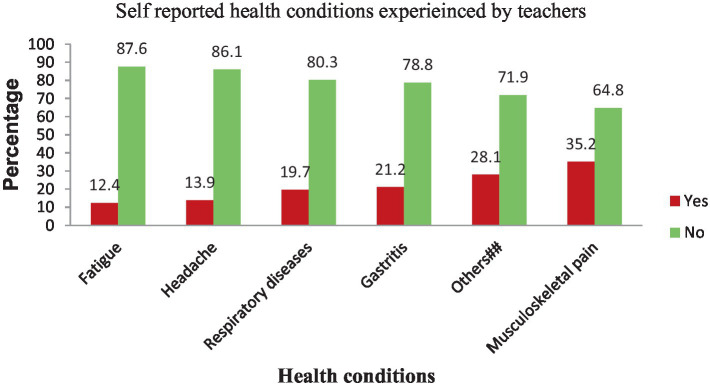
Bar chart showing causes of sickness Presenteeism among primary school teachers in Gondar city, Northwest Ethiopia, 2023 (*n* = 603). Keys: ^##^Depression, anemia, high blood pressure (HBP), kidney stones, heart problems, thyroid diseases, voice disorder, infection, insomnia/sleeping disorder, diabetes mellitus and dental problem.

Among the teachers who experienced sickness presenteeism, musculoskeletal pain was the most common health condition (35.2%), followed by gastroenteritis/stomach ulcers (21.2%) and respiratory diseases (19.7%) (Figure 3).

### Reported reasons for sickness presenteeism

**Figure 4:**
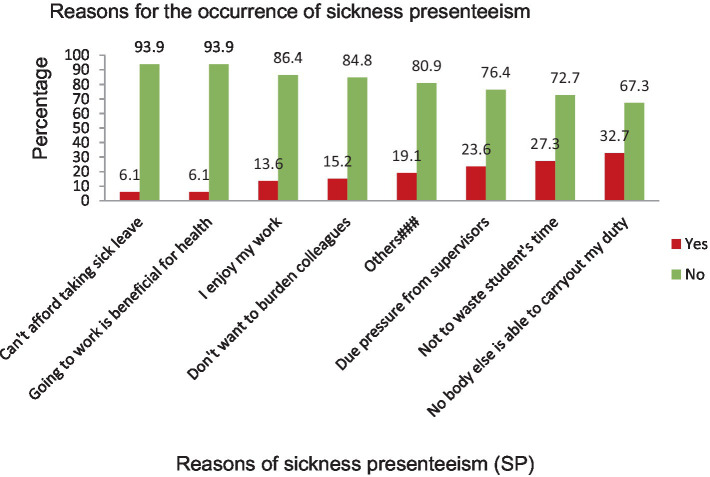
Bar chart showing reasons for sickness presenteeism among primary school teachers in Gondar city, Northwest Ethiopia, 2023 (*n* = 603). Keys: ^###^Putting pressure on you to come in, worried about being laid off, l wants to maintain my social network, do not want to be considered lazy, my pride depends on not taking sick leave.

The most common reasons for the occurrence of sickness presenteeism among primary school teachers were that nobody else was able to carry out my duty (32.7%), that they did not want to waste students’ time (27.3%), that they were under pressure from supervisors (23.6%), that they did not want to burden my colleagues (15.2%), and that enjoying their work (13.6%) contributed to their increased sick attendance in the study area (Figure 4).

### Factors associated with sickness presenteeism

Bivariable and multivariable logistic regression analyses were performed to identify the factors significantly associated with SP. Accordingly, school type, daily teaching hours, supervisor support, job stress, lack of staff replacement availability, colleague support, job control, teacher-student relationships, financial problems, household conditions, commitment, and attendance control were found to be the independent variables associated with SP in the bivariable binary logistic regression analysis, with *p* < 0.2. However, after controlling for confounding variables in the multivariable binary logistic regression analysis, private school type, low supervisor support, lack of staff replacement availability, low colleague support, unsuitable household conditions, and strict attendance control remained significant factors for the occurrence of SP (*p* < 0.05).

Teachers working in private schools were 2.21 times more likely to develop sickness presenteeism than teachers working in public schools [AOR: 2.21, 95% CI (1.14, 4.28)]. Teachers with low supervisor support were 1.53 times more likely to experience sickness presenteeism than their counterparts [AOR: 1.53, 95% CI (1.06, 2.20)]. The odds of developing sickness presenteeism were 2.74 times greater among teachers who lacked staff replacement availability than among those who had staff replacement availability [AOR: 2.74, 95% CI (1.85, 4.06)].

The probability of experiencing sickness presenteeism was 2.17 times greater among teachers with low colleague support than among those with high colleague support [AOR: 2.17, 95% CI (1.40, 3.37)]. The chance of experiencing sickness presenteeism was 1.5 times greater among those who had unsuitable household conditions for resting during illness than among those who had suitable household conditions for resting [AOR: 1.49, 95% CI (1.08, 2.34)]. Those teachers who had strict attendance control were 2.54 times more likely to experience sickness presenteeism than teachers who had less attendance control [AOR: 2.54, 95% CI (1.67, 3.85)] (Table 5).

**Table 5:** Bivariate and multivariable binary logistic regression analyses of factors associated with sickness presenteeism among primary school teachers, Northwest Ethiopia, 2023 (*n* = 603).

Characteristics	Categories	Sickness presenteeism SP (*n* = 603)		COR (95% CI)	AOR (95% CI)
		Yes (%)	No (%)		
School type	Private	123	44	3.09 (2.09, 4.58)***	2.21 (1.14, 4.28)*
	Public	207	229	1	1
Daily teaching hrs.	≤4 h.	201	216	1	1
	>4 h.	129	57	2.43 (1.69, 3.51)**	1.02 (0.54, 1.89)
Supervisor support	Low	179	113	1.68 (1.21, 2.32)**	1.53 (1.06, 2.20)*
	High	151	160	1	1
Job stress	Yes	139	86	1.58 (1.13, 2.22)**	1.16 (0.77, 1.75)
	No	191	187	1	1
Lack of replacement availability	Yes	202	83	3.61 (2.57, 5.08)***	2.74 (1.85, 4.06)***
	No	128	190	1	1
Job control	Low	160	103	1.55 (1.12, 2.15)**	0.98 (0.64, 1.54)
	High	170	170	1	1
Colleague support	Low	190	104	2.21 (1.59, 3.06)***	2.17 (1.40, 3.37)***
	High	140	169	1	1
Teacher-student relationship	Good	179	123	1.45 (1.05, 1.99)*	1.29 (0.87, 1.92)
	Poor	151	150	1	1
Financial problem	Yes	147	100	1.39 (1.01, 1.93)*	0.95 (0.64, 1.42)
	No	183	173	1	1
Over-commitment	Yes	128	82	1.48 (1.05,2.08)*	1.36 (0.89, 2.07)
	No	202	191	1	1
Household condition	Unsuitable	213	149	1.52 (1.09, 2.10)*	1.49 (1.08, 2.34)*
	Suitable	117	124	1	1
Attendance control	Yes	271	153	3.60 (2.49, 5.21)***	2.54 (1.67, 3.85)***
	No	59	120	1	1

Keys: 1 - reference category; AOR, adjusted odds ratio; CI, confidence interval; COR, crudes odds ratio; *statistically significant at *P* < 0.05; **statistically significant at *p* < 0.01, ***significant at *p* < 0.001, Hosmer and Lemeshow goodness of fit test *P*- value = 0.283.

## Discussion

Sickness presenteeism is a growing occupational health challenge for school teachers in developing countries, including Ethiopia, and is poorly recognized. Being able to work while sick can negatively influence teachers’ health and the quality of their teaching. Therefore, this study primarily aimed to investigate the prevalence and risk factors associated with sickness presenteeism in primary school teachers.

In the present study, the overall self-reported prevalence of sickness presenteeism in the last 12 months was 54.7%, [95% CI (50.9, 58.7%)]. This finding was in line with that of a study conducted in Germany (57.1%) (26) and Sweden (55%) (27). The prevalence underlines how much the teachers may feel pressured to work when sick to avoid being perceived as unreliable or weak. They may feel that they cannot take sick leave due to deadlines, fear of punishment, or a strong sense of duty to their students. This can lead to reluctance to take sick leave, as teachers may feel a strong sense of duty to their students and avoid letting them down.

The present study was in line with a study across occupations of sickness presenteeism among healthcare workers in western Ethiopia (52.6%) (41). This finding implies that despite professional differences, sickness presenteeism is an increasingly common public health problem in Ethiopia. Experiencing sick attendance in the workplace leads to employees’ loss of performance, delayed recovery from illness, and exhaustion (54).

Comparatively, the present study was greater than that in a study of Brazilian elementary school teachers (43%) (28). This might be because the teachers in the Ethiopian study were more likely to be female and younger and to have less experience than the teachers in the Brazilian study. These factors could contribute to higher rates of SP, as younger teachers may be less likely to take sick leave, and women may feel more pressure to come to work even when they are sick. Another possible explanation is that the availability of substitutes for teachers in Ethiopia and Brazil may differ, as they may face challenges in finding staff substitutes. This may result in teachers feeling pressure to work even when sick. Furthermore, teachers in Ethiopia lack support from colleagues, supervisors, or administrators, increasing their likelihood of working when sick.

The prevalence observed in the current study however was lower than a study conducted in Leon, Nicaragua (65.2%) (25). The discrepancy might be that Leon’s workplace culture may be more demanding, leading to increased stress and less sick leave. Individual differences, such as being organized and reliable, may also contribute to the difference in SP rates. Teachers in León may feel a strong sense of duty to their students and their jobs, increasing their likelihood of working when sick. Additionally, Leon’s heavy workload may cause teachers to become sick at work due to pressure to complete their work. Furthermore, teachers in León may have less effective coping strategies for dealing with illness (25).

According to the present study, the odds of developing sickness presenteeism among private primary school teachers were 2.21 times greater than those among public school teachers. This result was supported by the findings of a study of Portuguese elementary school teachers, which showed that private school teachers were 3 times more likely to experience SP than public school teachers (55). A possible explanation might be that private school teachers are under more pressure to meet difficult goals and attain high levels of achievement. This is because private schools often compete with each other for students, and they may have higher standards for their teachers. Leadership practices in private schools are more task-oriented and less employee-oriented than those in public schools. This is because private schools are often run like businesses, and the focus is on achieving results. This can lead to feelings among teachers that they are under pressure to perform and that their needs are not met. A teacher’s employment status in the private sector is employer-based. This means that they are not guaranteed a job and can be fired at any time (55, 56).

The present study revealed that teachers who reported low levels of supervisor support were more likely to report sickness presenteeism. This finding was supported by the findings of studies conducted in Germany (26) and Leon, Nicaragua (25). A possible explanation might be that teachers may feel that they have no other option or that they may be afraid of being penalized for taking sick leave. Teachers may not successfully negotiate days off due to poor communication or because they cannot demonstrate their health issues to their director, which motivates SP (50, 57). Another view is that less supportive supervisors may be more focused on the day-to-day operations of the school, such as ensuring that all lessons are taught and that students are learning, than on the health and well-being of their teachers. This can lead to a situation where teachers who are sick feel pressured to come to work, even if they are not feeling well. In addition, some supervisors may not fully understand the challenges that teachers face, such as working long hours, working a high workload, and dealing with students and parents. As a result, they may not take teachers’ health complaints seriously.

The current study showed that teachers who reported low levels of colleague support were 2.17 times more likely to report sickness presenteeism. This result was supported by the findings of studies conducted in Denmark (31) and Sweden (58). The explanation might be that teachers who feel unsupported by their colleagues may be more likely to come to work when they are sick. This is because they may feel like they have no other option. Unhelpful colleagues can increase the feeling that absence is unjustified.

According to the present findings, a lack of suitable households for rest was associated with sickness presenteeism. This result has also been observed in other studies (31). This observation might be explained first by the fact that staying at home when sick might be more stressful for teachers since their busy schedules prevent them from getting enough rest. Teachers could feel as though their homes are turning into their offices and vice versa. Second, teachers may feel bad about missing work even when they are ill since they may be enthusiastic about teaching and think that what they do is valuable to society (25, 27).

The present study revealed that lack of staff replacement availability was significantly related to sickness presenteeism. Due to the lack of substitutes, teachers in the study area had stringent work schedules with no room to take sick leave. This finding was supported by a study from Sweden (20). A possible explanation could be a lack of staff and a lack of professionals with multiple qualifications. There could be no one taking on the work of others other than those assigned for that particular job or fear of workloads when returning from illness. A more possible explanation might be that teachers with short staffing schedules do not want to burden their colleagues due to their sickness absence and because teachers feel responsible for and emotionally connected to their students. As a result, they want to minimize canceled lessons and go to school even if they are ill.

In this finding, strict attendance control was significantly associated with sickness presenteeism. This finding was supported by a study in Sweden in which attendance demands contributed to SP (59). A possible explanation might be that schools have strict attendance policies that require teachers to be present during working hours all day. Schools may also place much emphasis on attendance and tight deadlines. This can make it difficult for teachers to take time off, even when they are sick. School administrators may view perfect attendance as a badge of honor that can demonstrate signals to other staff. They may believe that teachers who have perfect attendance are more dedicated and hardworking. This can create even more pressure on teachers to come to work even when they are sick.

### Strengths and limitations of the study

This study is the first to investigate the prevalence and associated factors of sickness presenteeism among primary school teachers in Ethiopia, where the concept of occupational health is still less practical. Even though those occupational groups are at risk of presenteeism, there is no information on the status of these groups. Moreover, the study has limitations, as sickness presenteeism was measured via self-reports, which may have introduced recall bias and interpersonal differences in perceptions of illness concepts. Despite these limitations, we believe that the study provides a reasonably accurate assessment of sickness presenteeism and associated risk factors among primary school teachers in Gondar city, Northwestern Ethiopia.

## Conclusion

Sickness presenteeism is relatively high among primary school teachers in the study area compared to that reported in previous studies. The results of this study provide evidence that low colleague support, low supervisor support, strict attendance control, unsuitable household conditions, private school type, and lack of staff replacement availability are significantly associated with teachers’ sickness presenteeism. Therefore, it is recommended that schools should improve staff replacement availability, foster a supportive atmosphere, and establish connections with community groups for housing and financial assistance. Private school teachers can improve their support systems by regularly communicating with supervisors, providing mental health resources, and creating a supportive environment. Additionally, schools should review attendance control policies to ensure flexibility and consideration for employees’ health needs, such as paid sick leave and clear time off guidelines. In conclusion, although this research represents a first attempt at studying a phenomenon that has not yet been examined deeply in the Ethiopian context, there is a need for more studies to better understand the antecedents and consequences of presenteeism.

## Data availability statement

The original contributions presented in the study are included in the article/supplementary material, further inquiries can be directed to the corresponding author/s.

## Ethics statement

The studies involving humans were approved by the Institutional review board of the university of Gondar Institute of Public Health.PH Ref.No: IPH 2502/04/2023. The studies were conducted in accordance with the local legislation and institutional requirements. The participants provided their written informed consent to participate in this study.

## Author contributions

YM: Conceptualization, Formal analysis, Funding acquisition, Investigation, Methodology, Software, Supervision, Validation, Visualization, Writing – original draft, Writing – review & editing. DY: Conceptualization, Methodology, Supervision, Validation, Visualization, Writing – review & editing. MF: Methodology, Supervision, Visualization, Writing – review & editing. TA: Methodology, Supervision, Visualization, Writing – review & editing. CM: Methodology, Supervision, Visualization, Writing – review & editing. AB: Methodology, Supervision, Visualization, Writing – review & editing. AA: Methodology, Supervision, Visualization, Writing – review & editing. AT: Conceptualization, Methodology, Supervision, Validation, Visualization, Writing – review & editing.
